# Use of mobile health applications for health-promoting behavior among individuals with chronic medical conditions

**DOI:** 10.1177/2055207619882181

**Published:** 2019-10-10

**Authors:** Asos Mahmood, Satish Kedia, David K Wyant, SangNam Ahn, Soumitra S Bhuyan

**Affiliations:** 1 Division of Health Systems Management and Policy, School of Public Health, The University of Memphis, Memphis, TN, USA; 2 Division of Social and Behavioral Sciences, School of Public Health, The University of Memphis, Memphis, TN, USA; 3 The Jack C Massey Graduate School of Business, Belmont University, Nashville, TN, USA; 4 Edward J Bloustein School of Planning and Public Policy, Rutgers University, New Brunswick, NJ, USA

**Keywords:** Chronic disease, mHealth, smartphone, tablet, digital divide, health-promoting behavior

## Abstract

**Background:**

Chronic medical conditions (CCs) are leading causes of morbidity and mortality in the United States. Strategies to control CCs include targeting unhealthy behaviors, often through the use of patient empowerment tools, such as mobile health (mHealth) technology. However, no conclusive evidence exists that mHealth applications (apps) are effective among individuals with CCs for chronic disease self-management.

**Methods:**

We used data from the Health Information National Trends Survey (HINTS 5, Cycle 1, 2017). A sample of 1864 non-institutionalized US adults (≥18 years) who had a smartphone and/or a tablet computer and at least one CC was analyzed. Using multivariable logistic regressions, we assessed predisposing, enabling, and need predictors of three health-promoting behaviors (HPBs): tracking progress on a health-related goal, making a health-related decision, and health-related discussions with a care provider among smart device and mHealth apps owners.

**Results:**

Compared to those without mHealth apps, individuals with mHealth apps had significantly higher odds of using their smart devices to track progress on a health-related goal (adjusted odds ratio (aOR) 8.74, 95% confidence interval (CI): 5.66–13.50, *P *<* *.001), to make a health-related decision (aOR 1.77, 95% CI: 1.16–2.71, *P *<* *.01) and in health-related discussions with care providers (aOR 2.0, 95% CI: 1.26–3.19, *P *<* *.01). Other significant factors of at least one type of HPB among smart device and mHealth apps users were age, gender, education, occupational status, having a regular provider, and self-rated general health.

**Conclusion:**

mHealth apps are associated with increased rates of HPB among individuals with CCs. However, certain groups, like older adults, are most affected by a digital divide where they have lower access to mHealth apps and thus are not able to take advantage of these tools. Rigorous randomized clinical trials among various segments of the population and different health conditions are needed to establish the effectiveness of these mHealth apps. Healthcare providers should encourage validated mHealth apps for patients with CCs.

## Introduction

Chronic medical conditions (CCs) are leading causes of morbidity and mortality in the United States (US).^1^ About 60% of Americans have at least one CC, and 42% have two or more CCs.^2^ People with CCs have high healthcare utilization and are leading contributors to the excessive national healthcare expenditures.^1,2^ Individuals with CCs are at risk of impairment of daily activities due to a range of physical (e.g. having difficulties walking, climbing stairs), social (e.g. having trouble participating in family or social activities), and cognitive (e.g. having trouble with memory, making decisions) impairments.^2^ These impairments may significantly reduce individuals’ ability to control and manage their medical conditions.^3^ Evidence suggests CCs are further aggravated due to unhealthy diets and sedentary lifestyles.^4,5^

Many strategies to control CCs target unhealthy behaviors, often through the use of patient empowerment tools, such as mobile health (mHealth) technology. Prior studies have shown that mHealth applications (apps) using smartphones or tablets can empower those with CCs to manage their health.^6^ mHealth technologies offer the possibility of cost-effective, patient-centered tools to enhance individuals’ awareness of disease, improve disease tracking, increase adherence, promote healthy lifestyles, and induce positive behavior change.^7–9^ As of 2018, about 77% of the US population owned smartphones, an increase from 35% in 2011, and more than half (53%) of US adults owned tablet computers.^10^ The widespread distribution and increased ownership of smartphones and tablet computers has facilitated the rapid development of mHealth apps.^7^ More than 325,000 mHealth apps were available from various app stores, with an estimated 3.7 billion downloads in 2017,^11^ which is a dramatic increase from 200 million downloads in 2010.^12^ mHealth technologies have the potential to reengineer many facets of healthcare as access to the health information provided through mHealth and similar technologies has fewer temporal, geographical, and organizational barriers.^8,12^

Several systematic reviews examined the efficacy of mHealth apps in increasing physical activity,^13,14^ improving diet and nutrition,^14,15^ and improving health-related behaviors^16,17^ among the general population. For patients with CCs, there are a limited number of studies on the efficacy of mHealth apps in promoting health and health behavior.^3,7^ The published systematic reviews and meta-analysis in this area are mainly focused on mHealth-based interventions among individuals with diabetes.^18–23^ A few other reviews investigated the efficacy of mHealth-based interventions for a variety of CCs, such as chronic lung, cardiovascular, and cancer conditions.^3,24^ In general, mHealth apps seem to be moderately effective in the short run to help individuals self-manage their CCs, or to alleviate their symptoms.^3,20,22–24^ However, the long-term effectiveness of the mHealth apps at this point has not been well established, especially in chronic disease self-management. Moreover, we do not have evidence to support that desired outcomes of mHealth apps remain effective over long durations. In addition to these uncertainties, the use and level of adoption of mHealth among patients with CCs are underexamined. Despite these limitations, individuals with CCs are often the main target group for mHealth marketers.^25^

In this study, we seek to partly fill the gaps in the literature regarding mHealth apps use among individuals with CCs using data from Health Information National Trends Survey (HINTS). We use the conceptual model developed by Ronald Andersen and others which suggests that use of health and healthcare services by individuals is a function of their predispositions to use health services, factors that enable or impede such use, and people’s needs.^26–29^ One of the sets of factors in the conceptual model is predisposing factors which do not directly cause healthcare use but influence the likelihood that individuals will access healthcare. Predisposing factors include demographic factors, social structure factors, and beliefs including attitudes, values, and knowledge. The second group, enabling factors, includes individual level factors such as having a regular provider and income, and other factors such as individual’s urban/rural location. The third group, need factors, include both perceived needs (such as self-assessed health status) and evaluated needs (such as body mass index (BMI)).

Our aims are threefold: (a) To assess the association between having mHealth apps and using smart devices in health-promoting behaviors (HPBs) among individuals with CCs; (b) To investigate other predisposing, enabling, and need predictors of HPBs among smart device owners with CCs; and (c) To investigate the predisposing, enabling, and need predictors of using smart devices in HPB, specifically, among mHealth apps users with CCs.

## Methods

### Study design, settings, and sample size

Data for this study were drawn from the National Cancer Institute’s (NCI) HINTS (https://hints.cancer.gov/). HINTS is a nationally representative survey which has been administered every few years by the NCI since 2003.^30^ The survey targets civilian non-institutionalized adults 18 years and older in the US. It collects data on the public’s use of, access to, and needs for health-related information and health-related perceptions, knowledge, and behaviors.^31,32^ We extracted data from the HINTS 5, Cycle 1 (HINTS5-Cycle 1), conducted between 25 January 2017 and 5 May 2017. The survey had a unique two-stage sampling design. First, a stratified random sample of addresses (*n *=* *13,360) was selected from a file of residential addresses in the US. In the second stage, the Next Birthday Method was used to select one adult from each sampled household to receive the survey via mail. Overall, 3347 questionnaires were returned with a response rate of 32.4%. Other details of the survey are available online.^30^

**Figure 1.**
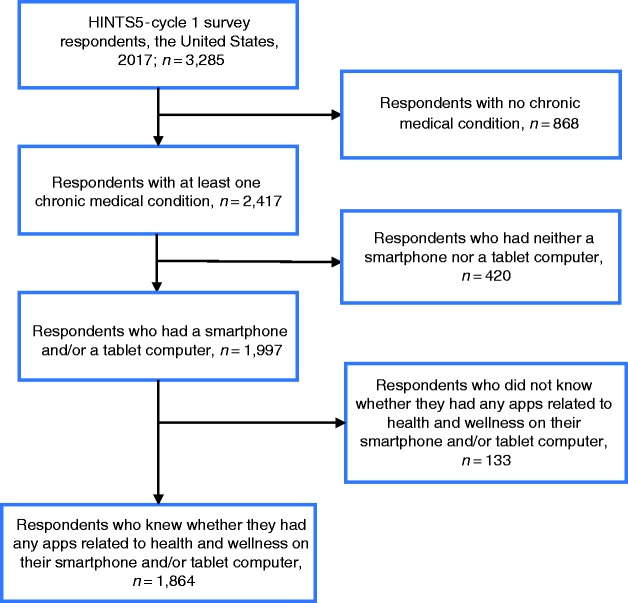
Flowchart illustrating the sample’s inclusion/exclusion process. HINTS5-Cycle 1: Health Information National Trends Survey 5, Cycle 1.

The initial unweighted sample size for HINTS5-Cycle 1 was 3285 eligible survey respondents. Since our focus was individuals who had CCs, we first excluded respondents without CCs. Survey participants were asked if they have been diagnosed for any of a list of CCs including diabetes, hypertension, heart conditions such as heart attack, angina, or congestive heart failure, chronic lung disease, asthma, emphysema or chronic bronchitis, arthritis or rheumatism, depression or anxiety disorder, and cancer. This step reduced the sample to 2417 individuals who reported having at least one CC. The rest of the steps of the inclusion/exclusion process are presented in Figure 1. The final analyzed sample represents 129.5 million civilian non-institutionalized adults 18 years and older in the US.

### Measurement of HPBs using smartphones or tablets

Information on HPBs among individuals with CCs was extracted from three survey questions in HINTS5-Cycle 1. Regarding achieving health-related goals, the respondents were asked: “Has your tablet or smartphone helped you track progress on a health-related goal such as quitting smoking, losing weight, or increasing physical activity?” Secondly, respondents were asked: “Has your tablet or smartphone helped you make a decision about how to treat an illness or condition?” Finally, respondents were asked: “Has your tablet or smartphone helped you in discussions with your healthcare provider?” These survey items were treated as binary dependent variables with responses of “yes” or “no.”

### Measurement of mobile health and wellness (mHealth) apps and models’ covariates

The variable that indicates whether an individual has an mHealth app follows from the question, “On your tablet or smartphone, do you have any ‘apps’ related to health and wellness?” This variable is treated as binary with responses of “yes” or “no.” We used this measure to calculate the proportion of the respondents with mHealth apps. Then in the multivariable logistic regressions, we used the mHealth indicator as one of the independent variables.

**Figure 2.**
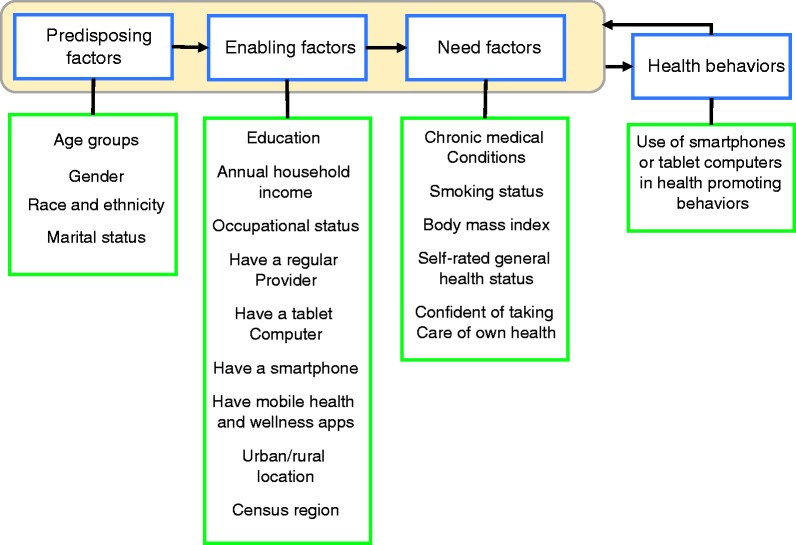
Conceptual framework to evaluate the relationships in our study.

In addition, we considered a series of variables that may be associated with outcome measures (HPBs). These variables also provided a pool of possible predictors in our regression models. The measures are similar to those in other studies that use the Andersen model. The pool included measures for individuals’ demographic variables (age, gender, race and ethnicity, marital status), socioeconomic variables (education, income, occupational status, urban/rural, census region), other enabling factors (having a regular care provider and ownership of tablets and smartphones), health behavior (smoking status), and health status (number of CCs, BMI, self-rated health status, and ability to care for own health). These measures were treated as categorical variables and were labeled as predisposing, enabling, and need predictors of HPBs. Figure 2 shows a conceptual model that includes these variables.

### Statistical analysis

The basic unit of analysis was individual survey respondents. We used SAS 9.4 statistical software (SAS Institute Inc., Cary, NC, 2014) to analyze the data. Final person weights and jackknife replicate weights from the dataset were used to estimate national level values and standard errors of estimates respectively.^32,33^ We used PROC SURVEYFREQ^34,35^ to generate weighted proportions, and the design-adjusted Satterthwaite Rao–Scott chi-square test^36,37^ was used to test for equal proportions in one-way analyses. To estimate the adjusted odds ratios (aORs), PROC SURVEYLOGISTIC^37,38^ was used to build multivariable logistic regression models with each HPB as a dependent outcome. To build the models, the entire pool of independent variables was initially incorporated in the models. Multicollinearities were checked in several steps of model building. The variables with the smallest contribution to the models were dropped with the backward elimination technique. Finally, the remaining potential predictors and interaction terms were incorporated into the models. The significance of the interaction terms was tested by the likelihood ratio test. The threshold for significance of analyses was set at *P*-value ≤.05.

## Results

### Smart device and mHealth apps users

In 2017, there were 1864 HINTS respondents (the “sample”) who reported having a smartphone and/or a tablet computer (“smart devices”) and having at least one CC. These respondents used their smart devices to track progress on a health-related goal (39.5%), to make a health-related decision (36.3%), and in health-related discussions with a care provider (34.2%) (data not shown in the tables). Table 1 shows characteristics of the respondents with smart devices and those with mHealth apps on their devices. Among those who indicated they have a smart device, 45.8% reported having mHealth apps. The largest subgroups for characteristics reported for the sample of 1864 device owners include 37.7% of respondents between 50 and 64 years of age, 52.7% female, 68.5% non-Hispanic White, and 62.2% married or living as married. Of these respondents, 32.1% had some college degree, 38.5% had an income of $75,000 or more, 59% were employed, 73.3% had regular care providers, 71.3% had tablets, 86% had smartphones, 84.2% resided in an urban location, and 38.4% were from the south. In this sample, 52.6% of the respondents had two or more CCs, 40.6% had a BMI ≥30, and 56.2% were never-smokers. Respondents most frequently self-rated their general health as excellent or very good (42.6%), and most frequently were very confident about their ability to take care of own health (45.4%). A similar proportional distribution exists among the 774 respondents who reported having mHealth apps on their smart devices (Table 1).

**Table 1.** Sociodemographic and other descriptive characteristics of the respondents (HINTS5-Cycle 1, United States, 2017).

	Have smartphones and/or tablet computers (*n *=* *1864)		Have mHealth apps (*n *=* *774)	
Variables	Weighted proportions (SE)	*P*-value	Weighted proportions (SE)	*P*-value
Have mHealth and wellness apps		0.025		
Yes	45.8 (1.89)			
No	54.2 (1.89)			
*Predisposing factors*				
Age groups (years)		<.001		<.001
18–34	15.1 (1.50)		22.6 (2.59)	
35–49	26.2 (1.91)		30.0 (2.48)	
50–64	37.7 (1.60)		34.1 (2.61)	
65+	21.0 (0.87)		13.3 (1.10)	
Gender		0.09		0.016
Male	47.3 (1.59)		43.2 (2.81)	
Female	52.7 (1.59)		56.8 (2.81)	
Race and ethnicity		<.001		<.001
Non-Hispanic White	68.5 (1.17)		70.2 (2.10)	
Non-Hispanic Black	11.8 (0.85)		11.8 (2.00)	
Hispanic	12.8 (0.90)		11.3 (1.58)	
Non-Hispanic Asian and others	6.9 (0.64)		6.7 (1.33)	
Marital status		<.001		<.001
Married/living as married	62.2 (1.64)		64.5 (2.83)	
Divorced/widowed/separated	14.2 (0.84)		10.1 (1.24)	
Single, never been married	23.6 (1.84)		25.4 (2.93)	
*Enabling factors*				
Education		<.001		<.001
Less than high school	9.3 (1.29)		5.6 (2.24)	
High school graduate	23.9 (1.66)		21.1 (2.75)	
Some college	32.1 (1.49)		30.2 (1.84)	
Bachelor’s degree	20.7 (1.00)		26.4 (2.17)	
Post-baccalaureate degree	14.0 (0.90)		16.7 (1.66)	
Annual household income		<.001		<.001
Less than $20,000	16.5 (1.20)		9.8 (2.10)	
$20,000 to < $35,000	10.6 (1.10)		7.5 (1.34)	
$35,000 to < $50,000	15.5 (1.41)		14.0 (1.93)	
$50,000 to < $75,000	18.9 (1.64)		21.1 (2.43)	
$75,000 or more	38.5 (2.16)		47.6 (2.83)	
Occupational status		<.001		<.001
Employed	59.0 (1.84)		66.0 (2.48)	
Others^a^	41.0 (1.84)		34.0 (2.48)	
Have a regular provider		<.001		<.001
Yes	73.3 (1.75)		76.8 (2.83)	
No	26.7 (1.75)		23.2 (2.83)	
Have a tablet computer^b^		<.001		<.001
Yes	71.3 (1.21)		79.5 (2.30)	
No	28.7 (1.21)		20.5 (2.30)	
Have a smartphone^c^		<.001		<.001
Yes	86.0 (1.09)		97.6 (0.81)	
No	14.0 (1.09)		2.4 (0.81)	
Urban/rural location		<.001		<.001
Urban	84.2 (1.39)		86.7 (2.50)	
Rural	15.8 (1.39)		13.3 (2.50)	
Census region		<.001		<.001
Northeast	18.3 (0.97)		16.4 (2.17)	
Midwest	22.0 (1.26)		19.6 (2.05)	
South	38.4 (1.33)		40.0 (2.13)	
West	21.3 (1.21)		24.0 (2.04)	
*Need factors*				
Chronic medical conditions		0.11		0.27
One	47.4 (1.60)		53.0 (2.70)	
≥Two	52.6 (1.60)		47.0 (2.70)	
Smoking status		<.001		<.001
Current	15.7 (1.45)		12.3 (1.70)	
Former	28.1 (1.31)		27.8 (2.30)	
Never	56.2 (1.68)		59.9 (2.43)	
Body mass index		<.001		<.001
Underweight (<18.5)	3.6 (0.64)		1.8 (0.50)	
Normal (18–24.9)	24.6 (1.74)		27.7 (2.61)	
Overweight (25–29.9)	31.2 (1.69)		28.7 (2.43)	
Obese (≥30)	40.6 (1.97)		41.8 (2.58)	
Self-rated general health status		<.001		<.001
Excellent or very good	42.6 (2.10)		51.0 (3.03)	
Good	37.3 (1.84)		34.6 (2.77)	
Fair or poor	20.1 (1.63)		14.4 (1.72)	
Confident of taking care of own health		<.001		<.001
Completely confident	21.2 (1.52)		23.5 (2.38)	
Very confident	45.4 (1.54)		47.8 (2.86)	
Somewhat confident	26.6 (1.83)		24.5 (2.93)	
A little confident/not confident at all	6.8 (1.20)		4.2 (1.17)	

Statistical analysis was carried out using the Rao–Scott chi-square test. *P*-values ≤.05 are statistically significant.

a “Others” category represents unemployed, homemaker, student, retired, disabled, and other.

b Such as iPad, Samsung Galaxy, Motorola Xoom, or Kindle Fire.

c Such as iPhone, android, Blackberry, or Windows phone.

app: application; HINTS5-Cycle 1: Health Information National Trends Survey 5, Cycle 1; mHealth: mobile health; SE: standard error of proportions.

### Factors associated with HPB among smart device owners

Table 2 exhibits the results from multivariable logistic regression models. The table provides findings regarding our first and second aims. First, respondents who reported having mHealth apps had higher odds of reporting that their devices helped them track progress on a health-related goal (aOR 8.74, 95% confidence interval (CI): 5.66–13.50, *P *<* *.001), make health-related decisions (aOR 1.77, 95% CI: 1.16–2.71, *P *<* *.01), and in discussions with their healthcare providers (aOR 2.0, 95% CI: 1.26–3.19, *P *<* *.01) when compared to those who reported not having mHealth apps on their devices. Second, other significant factors associated with using smart devices in HPB among respondents were age, gender, education, and occupational status. Older adults of ≥65 years had lower odds (aOR 0.24, 95% CI: 0.08–0.71, *P *<* *.05) of indicating that their devices helped them track progress on a health-related goal when compared to respondents aged 18–34 years. Females (aOR 1.55, 95% CI: 1.01–2.37, *P *<* *.05 versus males) and those who reported to be employed (aOR 1.97, 95% CI: 1.06–3.66, *P *<* *.05, versus others) had higher odds of indicating that their devices helped them track progress on a health-related goal. Older adults of ≥65 years had lower odds of reporting that their devices helped them make a health-related decision (aOR 0.38, 95% CI: 0.17–0.82, *P *<* *.05) and in discussions with their healthcare providers (aOR 0.50, 95% CI: 0.25–0.98, *P *<* *.05) when compared to respondents aged 18–34 years. Respondents with a post-baccalaureate degree had higher odds (aOR 2.68, 95% CI: 1.04–6.90, *P *<* *.05) of reporting that their devices helped them in discussions with their healthcare providers when compared to those with less than a high school degree (Table 2).

**Table 2.** Multivariable logistic regressions modeling the factors associated with health-promoting behavior among respondents with chronic conditions who have smartphones and/or tablet computers (HINTS5-Cycle 1, *n *=* *1864, United States, 2017).

	Model 1	Model 2	Model 3
	Tablet or smartphone helped track progress on a health-related goal such as quitting smoking, losing weight, or increasing physical activity	Tablet or smartphone helped make a decision about how to treat an illness or condition	Tablet or smartphone helped in discussions with healthcare provider
Variables	aOR (95% CI)	aOR (95% CI)	aOR (95% CI)
Have mobile health and wellness apps (ref: no)			
Yes	8.74 (5.66–13.50)***	1.77 (1.16–2.71)**	2.00 (1.26–3.19)**
*Predisposing factors*			
Age groups (years) (ref: 18–34)			
35–49	0.69 (0.25–1.90)	0.90 (0.44–1.85)	1.15 (0.65–2.02)
50–64	0.39 (0.15–1.02)	0.65 (0.33–1.29)	0.89 (0.42–1.89)
65+	0.24 (0.08–0.71)*	0.38 (0.17–0.82)*	0.50 (0.25–0.98)*
Gender (ref: male)			
Female	1.55 (1.01–2.37)*	1.32 (0.91–1.93)	1.04 (0.70–1.55)
Race and ethnicity (ref: non-Hispanic White)			
Non-Hispanic Black	1.18 (0.58–2.41)	1.47 (0.77–2.80)	1.26 (0.70–2.25)
Hispanic	1.75 (0.66–4.66)	1.03 (0.51–2.08)	0.88 (0.49–1.57)
Non-Hispanic Asian and others	2.34 (0.86–6.35)	1.18 (0.58–2.39)	1.64 (0.76–3.53)
*Enabling factors*			
Education (ref: less than high school)			
High school graduate	–	1.04 (0.36–3.03)	1.69 (0.66–4.31)
Some college	–	1.09 (0.35–3.36)	2.13 (0.80–5.67)
Bachelor’s degree	–	0.86 (0.28–2.62)	1.83 (0.69–4.86)
Post-baccalaureate degree	–	1.03 (0.31–3.44)	2.68 (1.04–6.90)*
Annual household income (ref: less than $20,000)			
$20,000 to < $35,000	0.92 (0.37–2.33)	–	–
$35,000 to < $50,000	1.26 (0.39–4.02)	–	–
$50,000 to < $75,000	1.51 (0.57–4.00)	–	–
$75,000 or more	1.71 (0.59–4.96)	–	–
Occupational status (ref: others^a^)			
Employed	1.97 (1.06–3.66)*	1.36 (0.77–2.39)	–
Have a regular provider (ref: no)			
Yes	–	1.34 (0.69–2.62)	1.64 (0.89–3.00)
Urban/rural location (ref: rural)			
Urban	0.67 (0.32–1.41)	1.63 (0.88–3.00)	–
*Need factors*			
Smoking status (ref: current)			
Former	1.50 (0.69–3.27)	–	–
Never	1.05 (0.49–2.25)	–	–
Body mass index (ref: obese)			
Underweight (<18.5)	–	1.61 (0.42–6.23)	–
Normal (18–24.9)	–	0.75 (0.48–1.17)	–
Overweight (25–29.9)	–	0.76 (0.47–1.21)	–

Statistical significance denoted as *P <* *.* *05, **P <* *.* *01 and ***P <* *.* *001.

^a^“Others” category represents unemployed, homemaker, student, retired, disabled, and other.

aOR: adjusted odds ratio; app: application; CI: confidence interval; HINTS5-Cycle 1: Health Information National Trends Survey 5, Cycle 1; ref: reference.

### Factors associated with HPB among mHealth apps users

Among those who reported having mHealth apps (*n *=* *774), education, occupational status, having a regular provider, and self-rated health status were the predictors of using devices in HPBs. Compared to respondents with education less than high school, respondents with a post-baccalaureate degree had higher odds (aOR 4.73, 95% CI: 1.18–18.89, *P *<* *.05) of using their devices in discussions with healthcare providers. Employed respondents had higher odds (aOR 2.35, 95% CI: 1.12–4.96, *P *<* *.05) of reporting their devices helped them track progress on a health-related goal when compared to those who were labeled under the “others” employment category (e.g. unemployed, homemaker, student, retired, disabled, and others). Employed respondents had almost two times higher odds (aOR 1.99, 95% CI: 1.06–3.75, *P *<* *.05) of indicating their devices helped make a health-related decision when compared to those in the “others” category (See Table 3). Compared to respondents with no regular provider, respondents who had a regular care provider (aOR 2.32, 95% CI: 1.16–4.65, *P *<* *.05) had higher odds of using smart devices in discussions with healthcare providers. Respondents who self-rated their general health as “good” had lower odds (aOR 0.36, 95% CI: 0.18–0.73, *P *<* *.01) of reporting their devices helped them track progress on a health-related goal when compared to those who self-rated their general health as “excellent or very good” (Table 3).

**Table 3.** Multivariable logistic regressions modeling the factors associated with health-promoting behavior among individuals with chronic conditions who have mHealth apps (HINTS5-Cycle 1, *n *=* *774, United States, 2017).

	Model 1	Model 2	Model 3
	Tablet or smartphone helped track progress on a health-related goal such as quitting smoking, losing weight, or increasing physical activity	Tablet or smartphone helped make a decision about how to treat an illness or condition	Tablet or smartphone helped in discussions with healthcare provider
Variables	aOR (95% CI)	aOR (95% CI)	aOR (95% CI)
*Predisposing factors*			
Age groups (years) (ref: 18–34)			
35–49	1.28 (0.42–3.90)	–	–
50–64	0.72 (0.24–2.14)	–	–
65+	0.54 (0.17–1.69)	–	–
Gender (ref: male)			
Female	1.57 (0.96–2.57)	–	–
Race and ethnicity (ref: non-Hispanic White)			
Non-Hispanic Black	–	1.70 (0.69–4.20)	1.87 (0.83–4.24)
Hispanic	–	1.41 (0.69–2.88)	1.58 (0.79–3.17)
Non-Hispanic Asian and others	–	0.89 (0.40–1.98)	1.13 (0.48–2.66)
Marital status (ref: single, never been married)			
Married/living as married	1.69 (0.71-4.03)	–	–
Divorced/widowed/separated	1.01 (0.35–2.91)	–	–
*Enabling factors*			
Education (ref: less than high school)			
High school graduate	–	2.46 (0.58–10.43)	3.47 (0.81–14.86)
Some college	–	1.58 (0.39–6.51)	2.73 (0.66–11.28)
Bachelor’s degree	–	1.44 (0.38–5.45)	2.72 (0.62–11.82)
Post-baccalaureate degree	–	2.56 (0.63–10.40)	4.73 (1.18–18.89)*
Annual household income (ref: less than $20,000)			
$20,000 to < $35,000	–	2.48 (0.69–8.99)	–
$35,000 to < $50,000	–	2.32 (0.68–7.97)	–
$50,000 to < $75,000	–	0.81 (0.27–2.44)	–
$75,000 or more	–	0.82 (0.27–2.51)	–
Occupational status (ref: others^a^)			
Employed	2.35 (1.12–4.96)*	1.99 (1.06–3.75)*	–
Have a regular provider (ref: no)			
Yes	–	1.58 (0.81–3.09)	2.32 (1.16–4.65)*
Census region (ref: west)			
Northeast	0.47 (0.22–1.01)	–	–
Midwest	0.99 (0.35–2.83)	–	–
South	0.52 (0.25–1.06)	–	–
*Need factors*			
Smoking status (ref: current)			
Former	1.14 (0.42–3.07)	1.68 (0.65–4.36)	–
Never	0.65 (0.23–1.80)	1.58 (0.66–3.79)	–
Body mass index (ref: obese)			
Underweight (<18.5)	1.90 (0.42–8.64)	–	0.78 (0.14–4.33)
Normal (18–24.9)	0.66 (0.32–1.36)	–	1.52 (0.81–2.85)
Overweight (25–29.9)	1.11 (0.50–2.44)	–	1.92 (0.94–3.93)
Self-rated general health status (ref: excellent or very good)			
Good	0.36 (0.18–0.73)**	–	0.75 (0.45–1.26)
Fair or poor	0.50 (0.20–1.24)	–	1.72 (0.86–3.45)

Statistical significance denoted as *P <* *.* *05 and **P <* *.* *01.

^a^“Others” category represents unemployed, homemaker, student, retired, disabled, and other.

aOR: adjusted odds ratio; app: application; CI: confidence interval; HINTS5-Cycle 1: Health Information National Trends Survey 5, Cycle 1; mHealth: mobile health; ref: reference.

## Discussion

In this study, we examined the association between having mHealth apps and using smart devices for health promotion among non-institutionalized adults with chronic conditions in the US. We also investigated other predisposing, enabling, and need predictors of HPB among smart device and mHealth apps users with CCs. Overall, those who had mHealth apps had higher odds of reporting that their smart devices helped them track progress on a health-related goal, make health-related decisions, and in discussions with their healthcare providers when compared to those without mHealth apps. Other significant factors associated with at least one type of HPB among device owners were age, gender, education, and occupational status. Among mHealth apps users, education, occupational status, having a regular provider, and self-rated general health were highly associated with at least one type of HPB.

Empirical studies similar to ours looked at the association between mHealth app use and specific health-related goals or behavior changes among a broader population of healthy and unhealthy individuals. For example, Carroll and colleagues,^39^ in a cross-sectional study, found that individuals who owned mHealth apps were more likely to report intentions to improve fruit and vegetable consumption, increase physical activity, and lose weight compared to those without mHealth apps. The same group of individuals were also more likely to meet recommendations for physical activity. In another study, individuals who were involved in physical activities for one or more times per week were more likely to report mHealth app download on their smart devices compared to those who reported never or rarely engaging in physical activity.^40^ In a survey of similar design in Germany, a positive association was reported between mHealth app use, increased physical activity, and consumption of low-fat diet.^41^

The higher prevalence of multiple CCs in older Americans and a lower rate of ownership of smart devices by older Americans may combine to create barriers for individuals with multiple CCs. Buttorff et al. report that between 2008 and 2014 the prevalence of multiple CCs in Americans over age 65 was constant at 81%, compared to 42% for all ages.^2^ In 2018, 46% of Americans over age 65 owned smartphones versus 77% for the population.^10^ This effect is magnified when we look at the rate of ownership of mHealth apps. Table 1 shows that individuals over age 65 comprised 21% of smartphone owners but only comprised 13.3% of those having mHealth apps. This contrasts with the age 18–34 group, which comprised 15.1% of the owners of smart devices and 22.6% of the owners of mHealth apps. This indicates that for even those individuals over 65 who have smart devices, the barriers to the ownership of mHealth apps are larger than the barriers for younger respondents. Cosco distinguishes between two levels of barriers for the elderly: access and adoption.^42^ Cosco notes that factors such as the cost of the phone may be a barrier to accessing the device, but the elderly may also be less likely to adopt some technologies even when they have the device. In addition, previous research found substantial gaps between the number of individuals acquiring an app and the number using the app.^43^ As a result there are a series of barriers that may include barriers to owning devices, barriers to acquiring apps once individuals own devices, and barriers to use of apps once individuals have acquired apps. For the ≥65-year age group, it is difficult to attribute a specific reason for each barrier, as there are multiple possible causes. For example, Cosco points out that the elderly may be more concerned with data security than are younger individuals.^42^ In terms of the Andersen model, this is an example of where some researchers distinguish between the factors that are “malleable” and those that are not. If policymakers view the problem as “age,” they cannot do anything about the individuals’ age. But if age is seen as a proxy for a bundle of other factors, one of them being concern for security, then policymakers have factors that they might target and change.

Given our findings in Table 1, it is not surprising that within the sample of 1864 smart device owners, those individuals aged ≥65 years were significantly less likely to be involved in each of the three HPBs. In other words, for the sample of smart devices owners the age variable was significantly related to each of the three HPBs; however, for the subsample of 774 individuals who have mHealth apps (Table 3), age was not a significant predictor of any of the three HPBs. The difference in results for the full sample and those using mHealth apps suggests barriers related to age may occur at the point where mHealth apps are disseminated, and the barriers are not due to a difference in the ability of older individuals to use the apps. It also could point to selection bias if individuals most comfortable with mHealth apps acquire them. This is an area that deserves further study. In terms of the Andersen model, the fact that age was not a significant predictor of any of the HPBs, for those having mHealth apps, suggests that the variables that made ownership less likely, including lower predisposition to use digital devices and being less enabled (e.g. fewer social contacts using apps), may be offset by greater need in the older group (as indicated by higher prevalence of multiple CCs). Regarding gender differences in HPBs, there are no definite explanations why females were more likely to be involved in HPBs using their smart devices. Other studies in the field have provided mixed results for gender in smart device and mHealth app use, and in associations between mHealth app use and HPBs.^39,41,43–45^

Among respondents who had mHealth apps in our study, level of education, occupational status, having a regular care provider, and self-rated general health status were associated with at least one type of HPBs. Bhuyan et al.^44^ investigated use of mHealth apps for the same set of HPBs among the general US adult population and found that among mHealth apps owners age, race, having a regular care provider, annual household income, BMI, self-rated general health status, and ability to take care of self were the significant factors associated with at least one type of the three HPBs. Specifically, they found that individuals of younger age, higher income and higher BMI were more likely to use mHealth apps for achieving health behavior goals. Participants who were young, African Americans, individuals who were completely or very confident about taking care of their health, and current smokers were more likely to use mHealth apps to help with medical care decision-making.^44^ They also reported that other factors such as being in older age groups, being African Americans, having a regular provider, self-rating general health status as good, and having a high BMI were significantly associated with using mHealth apps to ask a physician new questions or for a second opinion.^44^ Some similarities and differences can be observed when we compare our findings to Bhuyan and his colleagues. Our study was merely focused on respondents with CCs and we used a different modeling strategy to build our regression models. For example, among the mHealth apps users, age either was a weak, non-significant predictor of HPBs or had a minimal contribution to our regression models that was dropped out during model building steps. This could also mean that among mHealth apps users there were minimal age variations in HPBs compared to the significant role of age in HPBs in the whole sample of device owners.

The educational differences in HPBs among both smart device and mHealth apps users may reflect skills and confidence with the use of devices and possibly the social norms related to perceived value.^39^ Several studies attribute this positive association to health consciousness, health information orientation, and health literacy among smart device and mHealth app owners with higher levels of education.^46,47^ Education may provide a sense of efficacy, confidence, and knowledge to enable an individual to better navigate the health information provided through smart device and mHealth apps.^47^ It has also been argued that individuals with higher educational levels may be more likely to use alternative features such as social media and patient portals in health promotion, especially in communications with care providers.^48^ Reasons for higher odds of HPB among employees are less clear; however, it could be due to a shift in statistical power from respondents’ educational status or annual household income (which were alternatively dropped out) during model building. Sometimes, employment status can be used as a proxy measure and being employed represents a higher educational status or a secure income. Finally, having a regular source of care could mean that patients have potentially better access to health resources and health information to improve health literacy. In addition, the availability of a regular source of care may improve access to a range of digital health services, including health information from care providers.^48^ Hence, respondents with a usual care provider are more likely to be involved in HPBs.

Several insights associated with the Andersen model are applicable to our study. One extension of the Andersen model looks at differences between potential access and realized access. This concept recognizes that some people could access health services if they wished but choose not to access the services. In our case, there is a clear difference between potential access from owning smart devices and the realized access from pursuing HPBs with mHealth apps. Another extension of the Andersen model argues that equity exists when services are distributed based on need factors and not enabling factors. In our study, the finding that obese individuals had higher odds of having mHealth apps is an instance of individuals with greater health needs receiving more services, but findings for the income, health status, and age characteristics are all counter to the goal of serving those most in need. However, it is important to note that this assessment is relative. For example, although we found that higher income individuals have greater rates of mHealth apps ownership than lower income individuals, if any additional low-income individuals are receiving service through mHealth, then mHealth is providing access for the underserved. The same holds true for other groups, such as residents of rural areas.

Overall, our results show that a large group of individuals with CCs use smart devices and mHealth apps for HPBs. But the results also raise questions concerning the reasons a larger proportion of the individuals with smart devices are not realizing the potential benefits of mHealth. Clearly, the barriers to mHealth use are not all resolved by ownership of smart devices, as our results show “divides” in HPB among individuals with smart devices and among those with mHealth apps.

## Limitations

We have several limitations in our study. The HINTS is a cross-sectional survey of a nationally representative cohort of individuals, therefore we cannot infer causality and directions of the associations in the study cannot be indicated. In addition, there might be unmeasured confounding factors that potentially influence the results of our analysis. Such factors might have a correlation with smart device, mHealth app use, and the HPBs that we were unable to control for. For example, some confounding factors such as individual motivation, eHealth literacy and orientation, privacy and security concerns, readiness for change, self-efficacy, and health consciousness could influence download and use of mHealth apps.^43,45,46^ Hence, we were limited by the information provided in the HINTS dataset. In addition, survey respondent-related bias might have been introduced during the process of data collection. For example, respondents are liable for recall bias or misinterpretations of the self-administered questionnaire, or they might under/over report their CC status. Lastly, there is a large degree of variation in using the term “chronic disease,” and there are different classifications for CCs. For instance, some, but not all, classifications do not consider human immunodeficiency virus infection as a chronic disease.^49^ As another example, the World Health Organization and the Centers for Disease Control and Prevention classify chronic diseases differently.^49^ This might have influenced our sample selection and the overall interpretation of the results.

## Conclusions and future policy implications

mHealth apps are associated with increased rates of HPB among individuals with CCs. This is highly significant from a policy perspective, since nearly half of the respondents have mHealth apps, and since roughly a third of the respondents engage in each HPB. However, our results also suggest that the digital divide is influencing the distribution of the benefits of smart technology and mHealth. Some of the subgroups in American society with the highest incidences of CCs, for example older adults, appear to be most affected by a digital divide. This results in disparities in access to mHealth services for individuals with CCs. Efforts to increase mHealth participation for specific subgroups (i.e. elderly, rural, low income) might benefit from considering the magnitude of relevant barriers. As smartphones and tablets have spread rapidly, there is a major opportunity to transform the way healthcare is delivered. Smart devices and mHealth apps are expected to radically transform the practice and reach of healthcare and research.^8^ mHealth apps would have a noteworthy impact on patients with CCs, yet, to achieve the greatest levels of positive outcomes, we should overcome the barriers that currently exist. For example, improving usability may reduce the disparities in access and use of mHealth apps designed for patients with CCs, especially for older adults.^50^ Generally, current studies suggest that some features improve the use and effectiveness of apps including user-friendly design, less time consumption, individualized elements, real-time feedback, detailed information, and health professional involvement.^17^ Among patients with CCs, the ease of use of apps is a key factor motivating users to maintain engagement. mHealth apps that are designed to be simple, self-explanatory, and visually appealing have favorable usability feedback.^51^ Apps designed for patients with CCs need to be customizable to users’ needs and preferences to increase the level of adoption, motivation, and adherence.^51^ mHealth app developers should work together with healthcare professionals and researchers to design and deliver evidence-based apps that improve health outcomes and meet the needs and preferences of an aging society.^52^ Health policymakers and government agencies, such as the Food and Drug Administration, share the same responsibility to validate and review such technologies and smart medical devices to assure maximum benefit to patients. As the use of mHealth apps is expected to continue to increase in the foreseeable future, there is a need to further understand their effectiveness. Multidisciplinary teams should be brought together to develop theoretically sound mHealth apps. In addition, rigorous randomized clinical trials among various segments of the population with different health conditions are needed to establish the effectiveness of the mHealth apps. Meanwhile, healthcare providers can prescribe successful and validated mHealth apps for patients with CCs to increase use and maximize mHealth benefits for patients.
